# Morell's Inductive Mental Philosophy
**An Introduction to Mental Philosophy on the Inductive Method*. By J. D. Morell, A.M., LL.D. London: 1862.


**Published:** 1863-01

**Authors:** 


### Art. V.—MORELL'S INDUCTIVE MENTAL PHILOSOPHY.*

Dr. Morell is a clear seer in a twilight region, which is sometimes palpably obscure. He is a faithful and lucid narrator of what is disclosed in his observations; faithful even when his experience teaches and tells against his own convictions ; lucid in producing an exposition of what is in his view the genesis of thought, or of the laws by which the growth of thought is regulated; in a form which is at once recondite and attractive, and perhaps not the less so that his style is rich and redundant and imaginative ; equal to the requirements of the philosopher, but not above the grasp of even the partially educated thinker. He is, however, not only a clear but a comprehensive thinker ; he travels beyond the confines of his own consciousness, and, satisfied that a philosophy founded upon self-analysis, is not merely the portrait of an individual, but if unassociated with a consideration of psychical action wherever it is manifested and of the laws of those structures through which it is manifested—must be one-sided, he may be regarded as a physiological psychologist. As such only do we propose to deal with him in these pages.

From the fundamental distinctions of vital phenomena are the characteristic principle evolved in his present volume. He conceives that the attributes of vitality may be summed up in the resisting or repelling all that would destroy the entirety or oneness of the organism or the power of self-maintenance, in the selecting and appropriating what is conducive to life, or the power of attraction and assimilation. He recognises the same twofold law in operation in the property of the nerves of special sensation, in assimilating and propagating certain impulses from without, and then of exciting a reactive force which expends itself in motion communicated and in repulsion effected in reference to the world without. The instinct or power of adaptation to external circumstances, by which what is conducive to

* *An Introduction to Mental Philosophy on the Inductive Method.* By J. D. Morell, A.M., LL.D. London: 1862.

well-being is renewed, and what is noxious is repelled; and reasoning in separating and distinguishing as a necessary preliminary to the assimilation and complete adoption of truth—are held to be examples of the same law in the operations of mind-force.

We should be reluctant to affirm that there was both a play of fancy and an ambition to systematize in this identification of things which must still be regarded as distinct, however similar they may be rendered by description, when we have before us the doctrine of the correlation of forces and its influence even over logical minds. There is, however, a dogma at the very root of the supposed connexion of the laws of the general system and those of mentalization, which demands examination. "Were no impulse," says our author, p. 31, "to reach the mind within, we have no reason to think that any of its powers or capacities could be developed." This may be accepted as a declaration of faith in the creed of the sensational school, or it may not. If the profession be tantamount to an admission that powers and capacities, in other terms, mind, pre-exist the impulse from without and exist independently of it, but require such impulse for their growth and development, further discussion must embrace simply the nature, and condition, and relation of these powers and capacities. But if this formula contain the dogma that these powers, &c., must owe their existence and activity to the external activity, or, in fact, that the external impulse becomes, when impressed upon nervous tissue, the mind; or that it induces that molecular change which is supposed to occur either causatively or coincidentally with every mental act, there is then cast down the gage of battle in a strife which, as it is connected with a difference in mental constitution, must be perpetuated for ever. For this reason we are not disposed to accept the challenge, and moreover, Dr. Morell has, in various places, guarded himself, vaguely and loosely perhaps, against any accusation of scepticism of the independent existence of mind. It would, however, be unsatisfactory to leave the subject in the state in which it is placed by the quotation from the *Outlines of Mental Philosophy*. It appears to us that it can be demonstrated, that the mind exists anterior to impressions received through the special senses, or cœnæsthesis, and may be traced in the exercise of special powers and functions. We do not propose to reproduce either the history of the controversy as to innate powers, or to employ the weapons of those who rest content with the proposition of Leibnitz, Nihil est in intellectu quod non prius fuerit in sensu, nempe nisi intellectus ipse; or to repeat subtleties and refinements which, although they may contain, actually conceal truths, and which may well be dispensed with in an inquiry so practical, and in our eyes, so plain. Our illustrations shall be confined first to phenomena which appear to necessitate the pre-existence for which we contend; and secondly to the impossibility experienced by Dr. Morell, ingenious in phraseology although he occasionally is, in avoiding the employment of such a basis for many of his speculations. In "the preconscious or latent activities" (p. 11), which our author recognises almost in the same paragraph where he stigmatizes the notion that there are a certain number of separate, peculiar, independent faculties, lies the elementary nature of a faculty, a power, or capacity to do what is not yet done, which in the individual may never be done, and which differs from the thing done, or result, or manifestation. This must not be regarded as a mere potentiality, a mere latent force, which depends for activity upon extraneous circumstance, but as that condition of consciousness or mind (for these terms are now almost convertible,) which may exist in one species of animal and not in another; which differs in strength and permanency in the two sexes of the same species; which is not proportionate to other mental states, and which may not exist at all, yet leave the mind sane and entire, though limited in the nature and number of its applications; which differs at different ages; which may be in operation singly or conjointly; which descends from one generation to another; and which may remain healthy while the general intelligence is diseased, or become morbid while the general intelligence is vigorous and active.

The epoch during which intelligence becomes known to itself is at present a favourite resort of philosophers. We apprehend, however, that this preconscious period, as it is termed, is solely deserving of such a designation, because memory has failed to retain, or retains imperfectly, and as knowledge rather than as isolated ideas, the perceptions received, the sorrows experienced, the judgments formed; and that it may be made to yield up for our guidance vestiges which throw light upon the circumstances under which they originate.

It may be impossible to determine the precise moment at which the first conscious psychical act was performed, or to distinguish the results from those which may be referred to reflex action; but there is no difficulty in establishing a relation, in point of time, between an external impression and the phenomena which accompany it. Dr. Morell says, p. 126, that instinctive, nay, unconscious, reasoning processes take place within us from the earliest period of our history; that is, reasoning before, according to his theory, the law of reasoning or consciousness is developed.

We shall not advert here to the influence produced on the intelligence of offspring by the general condition of their parents, nor cite the instances where the child of the man temporarily deprived of reason by intoxication is an idiot, or where a similar result follows strong moral agitation, although these could be made to bear directly upon the investigation; but we would analyse the known connexion between the emotion of terror on the part of the mother during some stage of utero-gestation and the production of a timid, pusillanimous, terror-stricken child. This is not the propagation of constitutional qualities, for the parent may have been calm and courageous; it is not the dwarfing or curtailment of instinct or educability by an arrestment of development; but it is the transmission of a temporary powerful emotion from the mother to the fœtus, in which it remains a life-long and characteristic property, to appear whenever called forth by circumstances, and often when not so called forth. One case of this kind, such as that of Queen Mary and James I., is sufficient; but there are thousands suchlike. It must be observed that here the emotion alone of the mother is transmitted; neither the cause of the panic nor any of the attending circumstances, real or imaginary, could be so; or if they could, such a transmission would strengthen the view now advocated. But further, the feeling of the mother must have been experienced; must have reached and influenced some psychical condition in the child capable of being acted upon in a manner similar to that in which her mind was affected, of responding in some sense to the perturbation existing, and of retaining a tendency to activity under circumstances totally different. It is not necessary to pause to inquire whether the fear or the concomitant physical agitation of the mother led, or could lead, to any modification of structure in the nerve-matter of the fœtus, as such change must have been the consequence of the excitement lighted up by the feeling of fear in the fœtus, and the excitement of such a feeling shows that it must exist as a special faculty—as the sentiment of cautiousness—previous to the application of a specific stimulus. What may be called the physiology of an emotion has been selected in this instance as apparently free from the objections which may be urged, and unsusceptible of the various explanations which may be adduced, as to the undeveloped state and to the growth of the perceptive, the discriminative, and associating powers.

There is much instruction, however, upon this matter to be gathered even from a contemplation of the laws of hereditary descent. It is established beyond doubt that, however disturbed or marked by collateral influences, the same qualities may be traced from sire to son through several generations. This heritage embraces every psychical act, from the powers of generalization and abstraction, to the most trivial habit, the tone of the voice, or the arrangement of words.* This transmission is not confined to the natural and familiar operations of mind, but may be detected in the morbid and extravagant and exceptional manifestations. Were it a mere aptitude to imitate, a susceptibility to receive certain training or impressions, a solution of the process might be found in the recognised course of mental development; but when marked and distinctive perversity, when positive derangement of the same kind, when similar delusions reappear in succeeding generations, there is no alternative but to conclude that they have been derived from the same source, and that they are as much an inheritance as the form of the face and the colour of the hair. But to admit that the child is a mere duplicate, a reproduction of the corruptions and disorder of the nature of the father whom he may never have seen, who may have died before he was born, is to admit that not mere latent capacities, but that capacities in operation, although diseased operation, are in their full but depraved maturity transmissible. It is likewise significant that this descent goes on in defiance of all the antagonistic forces exerted by education, example, &c., and where the conditions supposed to be favourable to the creation of different dispositions, and the development of higher powers are confessedly and advisedly directed to this end. If it be true that for a century different members of the same race have been distinguished for oratorical powers, for eminent abilities in mathematics, arithmetic, or music, it is not enough that the general grasp of the intellect of these individuals was great and greater than other men; we cannot avoid the conviction that they shared some special gift in common, that this endowment was not the result of education, but inherent, and that it was no measure of general acuteness or judgment. The very defects by which certain races are characterised: the colour blindness of one, the defective powers of calculation of another, the poverty of language of a third; defects existing contemporaneously with intellectual superiority, would indicate, not that the mind was disintegrated or broken into fragments, but that, though placed in the usual relations to the external world, and although subjected to the process of development so well described by Dr. Morell, it failed to receive and recognise whole classes of phenomena, it did not possess the qualities exercised by other minds.

We shall not enter upon the vexed questions of the psychical differences between instinct and reason, nor as to that between instincts, instinctive appetites, and instinctive intelligence, nor as

---

* Morell, p. 361.

to the growth of sensation into instincts; although we hold that much error has arisen from confounding the thing done with the power to do it, and from confounding a want or a pain, first with the desire to get rid of it, or to satisfy it, and then with the powers exerted in the effort at emancipation or gratification. We are content to find instincts identical with those of the lower animals, and others closely approaching those of the lower animals, if not identical, acting in harmony with, or in subjection to, the highest best-regulated judgment; or solitarily and independently, as in certain classes of idiots and animalized tribes of human kind. We see these perpetuated through a long chain of individuals, both as propensities, mechanical tendencies, and what may be called intuitive capacities to discover. We see races lustful, bloodthirsty, constructive. It would be superfluous to insist upon the reproduction of such automatic acts as are mentioned by Dr. Morrell, (p. 360). We are assured that cretins build houses of the same shape and size from century to century; and that when more numerous than they now are, and living in a more primitive condition, they resisted brutishly attempts to change their habits. The bee affords an example of the transmission of various instincts. It is not germane to this subject to show that the insect was right without calculation, where the science of man failed; for we can form no conception of the process by which it is prompted to resort to particular forms, colours, &c., in carrying out particular ends. It suffices for our purpose that apart from any known effect of external impressions, or imitation, or instruction, a power to construct and a power to construct the same objects—a power which in man requires ratiocination—is transmitted, and appears in particular individuals of the race. We shall not quarrel with the hypothesis that the idea of a hexagonal cell with pyramidal base is stamped and stereotyped upon the consciousness or nerve-cells of the animal, because such permanence of impression is not in virtue of laws of similarity or difference, and must be anterior to experience of any kind. There is an error again in supposing that this is a solitary gift. Bees, the same bees, construct circular cells, and cells of various sizes, and modify their work as they approach or recede from external objects; their ordinary habits being subservient to the exercise of this function; and lastly, they knead with their mandibles the portions of the wax separated from between the scales of the abdomen into plates, which through the co-operation of several members of the community, are arranged according to a design which must be known to all, and according to which each must work in concert. It is not enough that they have inherited the notion of a particular form (and who shall assert that man has not done so, and of projecting it upon matter); we are called upon to confess that they possess a certain knowledge of different forms in relation to each other, of parts to a whole, and of working in relation to each other—but we shall not assert, in reference to the ultimate purpose of the preservation either of the species or of the individual.

The object of Dr. Morell's treatise is to accept what are designated the facts of the human mind, and to endeavour to reconstruct them synthetically so as to exhibit its structure in the natural, or, as it might be termed, the genetic order of growth. The author remarks (p. 340) :—" Just as the organs of the body are formed by the aggregation of cells, so the faculties of the mind are formed by the aggregation of residua." He proposes to himself to carry on the investigation inductively. The aggregation he conceives, is in the first instance due to the capacities of resisting and repelling, and of selecting: which are at once an attribute of vitality and a mental law. The point at which the vital diverge into the purely mental phenomena is in " the cranio-spinal system, which is constructed in reference to our converse with the outer world, containing a complete machinery for action and reaction between self and nature, the first movement of this system in carrying out its proper functions will be the first act of mind-force properly so called." (p. 42.) The primary step in the nexus is where impressions are received which do not pass into consciousness, but become latent, and which may appear or not, according to the presentation or the absence of the required stimulus. This involves much interesting speculation upon preconscious activity, which is roused and formulised into egoism and individuality by primordial pleasure or pain. Sensation is described as the effects of the external world upon nervous matter through the instrumentality of motion in the media. The dictum that we cannot recal a sensation must either be accepted with some reservation as to visual impressions, or the theory of residua without an actual deposit, here advocated, must be abandoned. The experience of many individuals, (it has been calculated two or three per cent.,) shows that in darkness or with the eyes shut, luminous and coloured images, new configurations, may be produced upon the retina, and form there vivid and distinct objects which can be retained under examination, and what is still more worthy of note, may be projected when the eyelids are raised, upon the opposite wall, and apparently seen there. We are not disposed to enter upon the inquiry whether such figures are actually thrown upon the retina by the mental excitement present, or are merely subjectively present; nor to urge the statement that Goethe and other men could by volition give the type for the phantasm, and that when the former closed his eyes and depressed his head he could cause the image of a flower to appear in the middle of the field of vision, which, however, did not for a moment retain its original form, but unfolded itself and developed from its interior new flowers formed of coloured, sometimes of green flowers. But the simplest phenomena of subjectivity, of bringing into consciousness perceptions which, although composed of residua never were presented in a similar arrangement, and are so new as to obtain no recognition, as to attract by their beauty, terrify by their hideousness, and in states of excitement to become external realities which haunt the steps of their creators, afford ample illustration upon this point. It is significant that although sensations may not be recoverable by volition, they may be produced and pass into the realm of perception, and subjectively, that is not through the ordinary channels of air, ether, &c. Electricity effects this; stimulating each special sense to the performance of its peculiar function; and connected intimately with this ramification of the subject are the facts that the blind recognise certain colours by touch; that the deaf and dumb are said to see concords upon the lips of a singer, and that they certainly detect harmonious sounds through the vibrations of the bones of the head.

Dr. Morell has particular views as to sensation. We sensize sound colour, according to him, by motions in the atmosphere or the universally diffused ether, or the parts of the body in contact with the special nerves. "It seems more probable that the flow of life through the body is accompanied with a constant thrill and movement in every part of the nervous system, so that the outward oscillations do not so much give rise to wholly new vibrations, as enter into conflict with the nervous action already going on, and give it that peculiar determination which is necessary to create any given sensation in the mind." (p. 74.) We accompany this transit of the phenomena of light, sound, &c., along the nerves without cavil, and the more cheerfully that we recognise our old friends the vibrations and vibrationuncles of Hartley, albeit somewhat modified, until we reach the nerve-pulp, and there we must pause. Every onward step is purely fanciful. There is not only no evidence of changes molecular or otherwise in the structure of the conducting nerve or recipient brain, but there is strong ground for believing the contrary. Our object, however, is not to controvert the theory of sensation, but to inquire into the process by which the sound, the colour, in whatever way conveyed, enters the domain of consciousness. It is obvious that whether a vibration, or a current, or galvanic force, it is a different thing before from what it is after it is sensized. A mere pulse of air, a rearrangement of the particles of a fluid, becomes a part of mentalization, not as a pulse, but is transmuted into a tone, may be recovered at will, may be by imitation communicated to others, and endures so long as the mind of which it is a constituent endures. If it be argued that the impression is received into consciousness, then consciousness, or the faculty of receiving and of being added to, must have preceded the reception. It is impossible to conceive that this first sensation was at once the impression received, the consciousness by which it was received, and the state through which it becomes a perception and a residuum. But again; the process of becoming a perception or subjective sensation is no *act* of consciousness, which is simply the state of mind existing for the time being. There must consequently be a faculty or channel by which sensation passes into thought, independent of and anterior to consciousness. Dr. Morell says, p. 41—"the outward impressions conveyed by the nerves are *elaborated* into ideas." Most readily is this granted: but by what means or process is this elaboration effected? The process cannot be inherent to the impressions themselves, it cannot be the property of the consciousness into which they are received, for the act by which they are cognised is consciousness. It must consist in intermediate powers, which receive colours, forms, or composite impressions in accordance with their special function; while the reception or rejection, the association or separation, constitute the laws of similarity, &c. of Dr. Morell.

But let us regard this in another light, and let us suppose the sound and colour and heat cognized; it is obligatory to show how these become connected together. It is not enough to say with Dr. Morell that there is a law of assimilation and repulsion. Such a law doubtless does exist, as well as an hundred others, and may regulate the growth of thought, but how does it explain the genesis? Montesquieu defines laws "as the necessary relations which spring from the nature of things." They are expressions of modes of action, not of the power by which the action is performed, and do not embrace nor solve the identification of the sound, the vision with the individuality, nor the association of these impressions, received through distinct media, with each other. This connexion cannot be the work of spontaneity in the impressions, nor of consciousness, which is passive; it must be an active operation, differing from all other mental states. But, lastly, we are told that these primary sensations rouse or constitute the Ego. That the impact of a mother's lip, or that a ray of light, may convey the first intimation of the Not me, is conceivable; but this conception involves three conditions: a receptivity on the part of the individual, one capacity to recognise the intimation as new or different fiom previous experience, and another capacity to appropriate into itself the perception of duality; processes which in germ contain all the elements of thought supposed to characterize the mature mind. We have spoken of experience antecedent to the first contact of the external air or of touch advisedly, and because we think that it is most unphilosophical, or, at all events, most unphysiological, to ignore the impressions received during uterine life, supplied both from the system of the mother and from the cœnæsthesis, and as the result of those structural changes which continue so long and which it is the purpose of gestation to complete.

It is imperative at this stage to consider the phenomena of a multitude of impressions derived from the special senses, entering into consciousness simultaneously or so suddenly as to be indistinguishable, and as rapidly as the glance which sweeps over a city at once. Is this crowd, this confusion of perceptions, if we may venture upon a visual illustration, separated and assorted by any quality inherent in each species or class? We cannot admit an elective affinity between the elements, allied to that in chemistry, nor a deposit according to a quality allied to gravity, nor arrangements depending upon force or temperature; yet if such alternatives be not accepted we must assume, not a law of relations, but a power by which relations are observed, and another by which related impressions are classified. It is not held that the suggestion of one idea by juxtaposition or similarity to another is a consequence of the position or resemblance; but because contiguity or similarity were perceived, recorded, and are recalled, or may recur as acts of the power or law of association. If we see a gaudy insect rest upon a flower, apparently sipping its sweets, and if ever after the sight or remembrance of the butterfly recal the rose, or the converse, there is nothing intrinsic to the one object or to the other, nor in their position in space, nor in the subjective union which connects them together, which can effect this; but some power which perceives, not the flower or the insect, but their associations, and nothing more, and which continues to do so as much after they have become faint perceptions or residua as when they were perceptions proper.

Perception, or the cognition of our sensational states, is referred by Dr. Morell to a change of state, or that alteration in the tissue of the brain which follows the replacement of one such state by another, and the blending of the first, "not entirely obliterated," with the second. "The primary sensation is not *entirely* obliterated; so far from that, its effect continues even while the consciousness is engaged with the new phenomenon B. This new phenomenon we thus see is a complex result, in which the experience of the first sensation is blended with that of the second, and a given mental effect is produced that differs materially from either of the two sensations taken separately and by themselves. The co-existence of the two mental changes, in fact, gives the first conditions on which an elementary and instinctive act of separation and comparison can be instituted; and this act forms the first link in the whole vast chain of mental development which ensues." (p. 83.) Now, it is palpable that the terms "blending together" mind and instinctive act, here signify something apart from both the impression and the material or molecular change which is supposed to cause or accompany it; they are, in fact, the intermedia or faculties in which and by which the impression is received, cognized, and incorporated with us. Although such impressions do not continue to exist in consciousness, they are supposed to be persistent and indestructible as residua, which are represented both as experience and as cells in the substance of the brain; where these are identical or similar they coalesce, and the multiplicity of our impressions is mingled together into combined images, and classified perceptive knowledge is the result. After investigating our perceptions of space, Dr. Morell attempts to demonstrate that the only "*essential* difference which can be set up between a perception and an idea is, that in the former case the actual object on which the mind is occupied is *present* to us, while in the latter it is *absent*." (p. 137.) A theory of attention is evolved from the action and reaction of ideas; where the occupation of consciousness is determined by the attractiveness or intensity of one or more, when they become composite, or when they present qualities in common. "The contest of ideas for the mastery closely resembles the relationship of forces as expounded in the science of dynamics. Here are two mental forces striving for the occupation of consciousness. If they are similar and act in the same direction, their results are combined. If they are opposed, then the one overcomes the other; but in doing so it loses a portion of its own power equivalent to that which it displaces. This is shown by the fact, that an idea is felt just so much the more vividly the less the mind is occupied at the time with other interests, &c." (p. 163.) The union of such elements insensibly leads to generalization; whereas their arrangement, similarity, contiguity in space constitute association. The theory of memory is given in the following propositions: "1, that there is no such thing as a separate and peculiar faculty; 2, that the possibility of memory is based upon the universal fact of the persistency of our mental impressions; 3, that as a voluntary reproduction of past phenomena it takes its start from the spontaneous classification of ideas involved in language; and 4, that its power depends upon the order and system which we consciously give to our ideas." (p. 214.) Imagination is understood to be "the faculty of creating and retaining images of things in the mind, of bringing them vividly into consciousness, and combining them into new forms." (p. 216.)

The proposition that every sane mind must pass through all the different forms of sensation and perception, or in other words, stages of development (p. 225), is not so demonstrable, if the history of the deaf and blind be kept in view. The testimony of those engaged in the education of the deaf and dumb, goes to show that, being thrown more upon their own resources, their pupils are even more thoughtful and reflecting than other children of the same age; which is tantamount to saying that the subjective processes are developed in an inverse ratio to the number of impressions received. But evidence exists that the mind grows up and appears to be possessed of the same powers, and to give manifestations much in the usual way, when the deprivation is much greater and when the mind or consciousness is shut in from the external world altogether. There was, and we believe still is, in the Massachusetts Institution for the Blind, an inmate, Laura Bridgeman, who has been blind, deaf and dumb, since she was about a year old. Her only knowledge from without must accordingly have been communicated through the senses of touch and taste. Our information as to her natural condition is defective; but after some years' training she is delineated as "much improved in personal appearance as well as in intellect; her countenance beams with intelligence, she is always active at study, work, or play, she never repines, and most of her time is gay and frolicsome. She is now very expert with her needle, she knits very easily, and can make twine bags and various fancy articles very prettily . . . . She is very docile, has a quick sense of propriety, dresses herself with great neatness, and is always correct in her deportment. She can speak by means of the manual alphabet with great facility and rapidity; her vocabulary comprehends the names of all common objects; she uses adjectives expressive of positive qualities, such as hard, soft, sweet, sour; verbs expressive of action, such as give, take, ride, run, in the present, past, and future tenses. She connects adjectives and nouns to express their qualities; she introduces verbs into sentences and connects them by conjunctions, &c. In short, it would be difficult to find a child in possession of all her senses and the advantages that wealth and parental love can bestow, who is more contented and cheerful, or to whom existence seems a greater blessing than it does to this bereaved creature, for whom the sun has no light, the air no sound, and the flowers no colour nor smell." Regarding this narrative merely from a physiological point of view, there are qualities described, and the whole mental machinery in motion, in utter default of the inductive method of growth; impoverished it must be in what has been received, contracted in the scope of application, but energetic and active as functions of the cerebrum. But taking one step further in the same direction, we have seen the blind, deaf and dumb, partial idiot. This was an instance not of idiocy by deprivation, an *enfant arrière* in consequence of the shutting up the windows of the soul. There was congenital physical deformity, as well as mental defect. And yet this creature pawed the air, not merely in search of the *not me*, but for companionship, for a particular companion; and having found and fondled its nurse, was satisfied. We deny that touch, in conjunction with the external world, does more than intimate a sensation which does not place us in any relation to the thing touching us, which cannot be distinguished from the heat or cold by which it is accompanied, unless there be a pre-existing and presiding power, which compares and distinguishes and arrives at a conclusion. But let it be supposed that the idiot consciousness has been apprised by the instrumentality of these media that there is something outside, beyond, independent of itself; and let it be supposed that smell may have extended this apprehension by suggesting that Nurse A. is different from Nurse B., how does it arise, unless there be an *à priori* substratum, that there is a seeking for Nurse A., a preference for Nurse A., and satisfaction and repose in the presence of Nurse A.?

We are not disposed to dispute the doctrine of a concept which, however, seems identical with that of typal perceptions and generalized ideas. "A vast number of objects are constantly presenting themselves to the senses; the perceptions of the objects leave residua in the mind; these residua insensibly blend together by the law of similarity; and when a result is obtained in which all reference to time and place is lost, and all memory of the actual objects of perception from which the generalization takes place, we term the result a *concept*." (p. 236.) Unless a substratum, be it faculty or receptivity, be admitted, every theory of mind becomes at some, and generally the initial stage, impracticable, and the obstacle is as great in the analysis of conception as of perception. If we are compelled to suppose that the thing sensized, on coming into consciousness, becomes the mind, we encounter two difficulties: that of the mind contemplating itself; and that, as we have elsewhere insisted, of any other than material media, in or by which a condition of nerve fibre passes into a perception. Let us suppose the first occasion upon which the finger is carried around an orange or the edge of a piece of money, for the process is the same, and conveys to the brain a series of impressions and vibrations, which from that time and ever subsequently, and irrespectively of the circumstances under which they were received, serve to represent a circle, we cannot avoid holding, either that there is something in these pulses when reaching consciousness, which constitute the idea of a circle, and which for the time being is the mind, that is, the state perceived being the state perceiving, which is absurd; or that the blending together of the residua and the formation of a concept, must take place out of consciousness, as we have no knowledge of such coalescence, which is untenable on the hypothesis; or we must have recourse to the belief that these conditions are cognized by a faculty or receptivity, specially endowed with such a function, and which presents them anew, and as often as they are recollected, or willed, or compared, as the case may be. There is yet another difficulty. Let us suppose that contemporaneously with the motion of the finger, the eye transmitted a sensation, subsequently called yellowness, and that the olfactory nerves were impressed with a perfume; in what manner can these qualities be associated together so as to suggest the complex notion of an orange; is it by a mere law of blending, and without that power or "classification" (p. 157) which unites impressions received through different sense-channels into a whole in the mind and ever after recognises it as a whole, not in virtue of comparison with different similar bodies, but as the whole originally perceived. It will not suffice to affirm that the perception results from the change from one impression to another, for this supposes a succession of phenomena which may not exist and suggests this other dilemma, that if there be not specific and independent faculties, why should one impression displace another, a colour supplant a form, in consciousness; and moreover, how can we arrive at the conception of one form by comparing it with another (p. 234), seeing that the idea either of a circle or of a quadrilateral, as the case may be, must have preceded the other in order of time, and been as perfect before as after comparison; and lastly, by what process is the act of comparison effected.

But in this process of development, it may be fairly questioned, if the growth depends upon the operation of laws such as have been described, and upon the frequency of their exercise, why should there exist ultimately any difference in the mental condition of individuals placed, or who may be placed, as nearly as may be, in similar circumstances and subjected to the same training. The difference in culture may explain to a certain extent, the great diversity observed in the manifestation, but not in the intensity and grasp of the intellectual powers, as well as in the passions and sentiments. Dr. Morell has admitted (p. 561),

"that the primary individuality with which we are born has *something* to do in the formation of the character is still true, for it is this individuality which gives a bent to our mental sympathies, and thus greatly contributes to determine the course in which our faculties operate, as they are in process of formation; to determine, therefore, the *kind* of experiences which we amass on the road;" a passage tantamount to a confession that individualization involves all mental powers. There is no reason for thinking that in the idiotic and imbecile mind, where the frame is natural and healthy, the laws are different from those in the most manly and mature intelligence, or that within a certain range they act with less precision or accuracy. But this certain range implies the absence of capacities and aptitudes, perhaps the prominence of others, and *de facto* a rudimentary substratum, which, though acting in the same mode, can act only in certain directions; which cannot grow into maturity because the faculties by which the mind receives, co-acts, and builds up and applies the impressions which are poured in from without in the same profusion, in the same order, and with the same appropriateness, as in the case of the powerful and capacious understanding, does not exist. It is interesting, that whatever the number of impressions in such cases, the residua are few and imperfect. An illustration is thus afforded where the conditions of mental growth are the same, but where dwarfing, stuntedness, and deformity, perhaps, accrue, not from interference with the laws of development, but from the state of a substratum of which these laws are modes of action. We are betrayed by a word. In speaking of the growth of mind there is, perhaps, presented to the imagination, if not to the apprehension of every man, an enlargement, an expansion, if not in space, in some vague amplification of capacity. When the apparently idealess mass, budding in the cradle, after a lapse of years, and a sensible alteration in structure, size, aspect, is compared with the philosopher, the astute merchant, the sagacious workman, it is natural to conceive that the qualities which so conspicuously distinguish the mature man, have grown with growth and strengthened with strength. This is true in a sense, but only in the sense of the relation of objectivity. Without going into the fanciful correspondence which may, however, exist between the world within and the world without, nor adducing the adaptation of the mental condition to the impressions which it receives, it is obvious that the nature of the mental operations do not alter through use or time. The perception of individuals and genera is as distinct, the comparison of two propositions is as completely effected, the feeling of joy, and ambition, and anger are as genuine in youth as in manhood; just as the first act of respiration or the first sensation of pain are the same as the last. These operations may in mid-life be more perfectly performed, or, it may be, modified, by previous experience, or observation, or habit; but these changes are not resolvable into growth. It is true that all our aptitudes do not come into activity at once; but this is determined by the changes in organization, and by the influence produced on that organization by external agents. The multitude of ingesta crowded in upon consciousness without order or sequence, or according to a succession established by the mind itself, amounts to nothing more at the end of a long life than materials for thought; the copiousness of illustration obtained by accident or by cultivation and actual search, afford manifestation of the mental states; but these states remain in character as if they had been stunted and starved by a limited range of observation; and they have added only to the intellectual stature only so far as they have multiplied the relations of powers with external impressions or between themselves, and have imparted by repeated contemplation the habits of familiarity and facility. If this be a correct exposition, the laws or relations between the states of mind must at all times be the same; and there is as great a difficulty in conceiving growth in time, as in space, if these states are involved, as they must be, in the very first operations which are cognisable or analysable by others; and, whether such laws arise from the blending, or separation, or accumulation of residua, or between the states of mind to which such residua are addressed, they must come into operation, not by gradual succession and laborious aggregation, but at once and at first. We shall not adduce in support of such a theme that discoveries and great efforts and achievements are the work of young men; but there is an element calling for consideration in the fact that, in cases of precocity the apparent growth anticipates the pabulum, the nourishment from without by which it is supposed to be carried on, and that in all men the first phase gives tone and form to the subsequent life; that the boy is not merely figuratively father of the man, but that the fully evolved being and life is a mere repetition of an early or the earliest impress.

We would not demur to designate that mental procedure judgment, which assigns any individual object to its proper class, or any given species to its proper genus, although these operations appear in nature identical with the law of perception, and suggest the difficulty that there must be a process of this kind applied to all ideas; and yet it cannot be affirmed that we judge of the colours of metals in the same way that we judge of moral qualities.

Reason is described (p. 289) as "the co-ordinating power in relation to all our other intellectual processes—as that which gives unity and solidarity to them, aiding us at once in the pursuit of truth, and in adapting our lives to the state of things in which we exist;" and at p. 336, as "the power of co-ordinating all the other intellectual processes, so as to give rise to *human convictions*, and enable us to adapt ourselves to the universe in which we live. Of these convictions, the first and most important are those which rest upon indubitable *objective* grounds, and which, therefore, we term *knowledge par excellence.* Those convictions which rest upon universal consent, but which can produce no objective proofs, we term natural beliefs. While, lastly, those which rest on the subjective impulses and promptings of our individual nature, are to be considered only in the light of personal convictions." There is less perspicuity in the analysis of Will. We cannot accept, as a basis of expiscation, "just as the organs of the body are formed by the aggregation of cells, so the faculties of the mind are formed by the aggregation of residua; both the one and the other, however, starting at first with a definite individuality, and with the possession of primordial powers and susceptibilities corresponding with the elements of nature, in the midst of which we are placed." Nor, "Just as we have traced the development of the understanding and reason through a succession of stages, beginning with the primordial instincts, and rising up successively to more and more complicated forms of intelligence, so ought we now to trace the development of the will, through a succession of similar steps, from the first instinctive *efforts* of our nature, up to enlightened and self-regulated activity. Moreover, we should naturally look for the same great fundamental laws to regulate the growth of the will as we have seen regulating the growth of the intellect, I mean the laws of combination and separation. The difference in the application of these laws, however, will be this, that whereas we had to do before with the combination and separation of ideas, we have now to do with the composition and resolution of mental forces. Thus, we are brought into a sphere of mental dynamics, the primordial impulse of which, indeed, like that of the universe, is transcendental (*i.e.*, lying beyond the possible reach of human experience), but of which the real manifestations and developments are quite capable of being analysed and tabulated." It would appear from this that our author believes that we are "brought into a sphere of mental dynamics, the primordial impulse of which is transcendental," &c., but yet he states (p. 360): "To speak of a *primordial innate instinct* for art, music, or mechanical contrivance, would of course be absurd. If such instincts formed a part of the essence of human nature, they would be universal, and would not be confined to a select few." Yet how to account for this instinct in its highest degree in idiots, and those so situate as to be removed from example and instruction, is next to impossible. But besides this impulse, the complex nature of the human will, in its developed state, is said to consist of four elements: "1, the intelligence to comprehend a purpose, and plan a course of action leading to it; 2, the capacity of balancing motives; 3, the power of decision; 4, the motor mechanism, standing at the soul's behest, by which the decision can be carried out. Then, at last, there may be an act which can be called purely volitional. (p. 367.) We conceive that Dr. Morell has been hampered, by associating together, as the same thing, will as exercised objectively and will as exercised subjectively. He says (p. 368), "The only element *peculiar* to it, the will, is the active or motor power developed through the nervous organization; the volitional use of this power being wholly due to the co-operation of the intellectual faculties." Now the most perfect volitional acts are performed, not merely without the co-operation of the muscular apparatus, or in resisting the tendency to move; but in regulating a course of thought or feeling, or in concentrating the attention to the exclusion of all consciousness of external objects and organs altogether. The paralytic wills to raise his arm, and the mental act is as perfect and well purposed as if the muscles obeyed the injunction. Is there not, in short, a broad distinction between volitions affecting ratiocination and movement? do we not obey the dominant faculty, or force, whether it determine repose or action? is there not a will in every faculty? and lastly, is there not a hiatus between the inclination and the resolution, which is not necessarily filled up by a purpose, nor by an equilibrium of motives, but by will itself? Will is something different from the desire to do a particular act, but it may proceed upon a resolution or a sense of duty in direct antagonism to that desire; it may resist the desire, which may continue, although an opposite course to gratification is determined, and it differs from the resolution which is the result of a mental struggle or process, and may not represent the desire.

In the psychology of the feelings the fundamental distinction of an idea from a feeling need not be entered upon, nor the position which the former holds between intellect and will; but when it is laid down as an axiom that if we had no ideas we could have no emotions, we must appeal from the well to the ill-regulated, from the sound to the unsound mind, in which pride and terror and wonder arise as conditions of consciousness without corresponding ideas, and where the whole being may be absorbed in a sense of despair. And when emotion is defined (p. 414) as "a tension of our ideas," *i.e.*, depending on the special mode in which the residua affect each other and pass in and out of consciousness, we inquire, is this tension resident in the structures with which feeling is associated, or "in the irregular and extraordinary flow of the *mental life* of the current of ideas through the consciousness," or "the consciousness which causes a rapid flow of ideas through the mind, a struggle and tension in our thoughts, which appears in the form of some emotion?" &c. (p. 413.) We are not captious; we are not hypercritical; we know the poverty of language and the necessity for employing familiar images to convey profound and unfamiliar conceptions; we are ourselves often guilty of materializing abstract thought; but here we must complain, first, that ideas are depicted as flowing through *consciousness;* secondly, that consciousness is accused of causing a flow of ideas *through* the *mind;* thirdly, that this tension, in any sense, is unknown to physiology or psychology; fourthly, that not only is there tension but also a struggle, or two factors, or alternate tension and relaxation, assigned as the cause of some emotion, "it may be grief, despair," &c., the precise nature of which is determined by the particular interests which are disturbed. It would be easy to show that Dr. Morell has been a victim here to fancy, a loose analogy, or the ambition of solving every question; but the only importance attachable to so playful a disquisition is in the temerity of making all emotions, whether high pitched or low, pleasurable or painful, depending upon ourselves or others, result from the same unknown quality of ideas; in utter disregard of the facts that in many instances the feelings are the factors; that in other cases they depend exclusively upon bodily, even morbid conditions, not directly connected with the nervous system, and that they are apparently as frequent and powerful in what are called preconscious states where the existence of ideas is incapable of demonstration. But Dr. Morell does treat of feelings as independent of special ideas, which appear to be congeneric with those previously discussed. These are enumerated at p. 430; but though the origin of the feeling is referred to organization, its course is again clogged by the theory of "creating a tension in the tone of our conscious life." One further advance, and we find our author abandoning his favourite inductive method, out-platonizing the wildest disciples of Plato, proclaiming something more advanced than even the immutable and eternal forms: "It seems impossible (he says) that mere outward and passive forms should be able to stimulate the flow of our ideas unless these forms expressed ideas themselves. An outward object, with or without meaning, might produce *sensations*, and thus stimulate the nervous system; but to awaken the *mind*, to stimulate the flow of *ideas*, and kindle a purely *mental emotion*, (the beautiful,) there must be something kindred to mind in the object—there must, in fact, be reason visibly embodied in form or audibly embodied in tones." (p. 147.) If there be not in these words both an admission of a substratum, the state, the "something" in the mind "kindred" to the outward form by which it is stimulated, and of the expediency of employing deductive reasoning, it is difficult to conjecture what the meaning or aim of the passage is.

No one can attentively peruse this work and fail to observe, that although the author commences with the declaration, (pp. 9, 11), that the notion of there being a certain number of separate individual faculties has stood more in the way of a purely inductive treatment of the mental phenomena than anything else, how frequently in the course of his inquiry he is compelled to assume or to write as if he assumed the existence of a mind of which the states were faculties. This is less the result of the poverty of language than of the necessities of the subject. We have first (p. 36) "the nascent spark of intelligence in the primary cell, *the soul* in its primary unconscious state." We might claim the concession of the whole postulate from the extraordinary powers conferred upon this spark of divine intelligence, when (p. 37) it is represented as "adding cell to cell, shaping the tissues into organs and limbs, adapting the body to perform the functions of life, constructing the wondrous network of the nervous system, and giving it power to vibrate at the bidding of the world without;" or when (p. 126) "instinctive reasoning" processes—nay, unconscious reasoning processes—are said to take place within us from the earliest periods of our history; and it is no less wonderful that the soul should adapt the bodily organism to its own future uses, which we know it does, than that it should tacitly build up its own knowledge of that world from the phenomena presented." We shall not designate this as a dream at the dawn of philosophy, for we know it to be Hegelism, and that it forms a leading feature of many recent works. Nor shall we speak of the psychologist's plagiarism from the poet's dogma—

> "There's a divinity within us
> That moulds our wills, rough-hew them as we may."

But we may remark that cell formation would appear, from the tendency of recent observation, to be under the influence of physical laws, and that it may be imitated chemically. It may be demanded further, what corroboration is there of spontaneity to be found in the form or metamorphosis of cells? It would be a struggle with phantasmagoria to combat such a speculation by the physiological objection that such complex functions cannot be performed by a number of isolated cells; that these cells are constantly perishing, transforming, changing into cartilages, vessels, nerves; that when structure is known to be connected with psychical action, cells are broken down, and the tubes resulting from their rearrangement contain deposits on which their peculiar properties seem to depend, are formed at a subsequent time.* The hypothesis advanced is either an expression of the truth that there is a harmony between the shape and structure of organs and the purposes which they subserve, which is universally admitted; or that organization results from one of its own qualities, or one of those with which it is associated, that the cell is self-developing, that we are our own creators; or it attributes to the rudimentary stages of consciousness what cannot be effected by that mental condition in its ultimate power and strength. In fact, all direct operations of consciousness, such as attention, interfere with nutrition and formative growth; and this may explain why the processes by which the frame is originally built up precede conscious direction of thought to existing conditions, as well as introspection and anxiety, inasmuch as these, in place of shaping and guiding to salutary increase or other modifications, would induce disorder and disease. Moreover, in all instances where the cœnæsthesis imparts distinguishable intimations, they are significant of disorganization and decay.

We next find that outward impressions conveyed by the nerves are elaborated into ideas (p. 41), that tendencies or capacities, not original, but so elaborated, become hereditary; and further, that a *latent* intelligence within us works teleologically, apart from will, feeling, sensation, or any kind of consciousness whatever. (p. 52.) In treating of the indestructibility of perceptions, the mind at birth is described as possessing neither ideas nor faculties, but only a "germinal nature." (p. 89.) Yet in a few paragraphs a perception is asserted *not* to continue in consciousness, but still it remains in such cases "tacitly in the mind," that it may again be brought into consciousness by any sufficiently active suggestion. Now, if not preserved in consciousness, what is this "mind," in which it is said tacitly to exist? or admitting that these residua are "left in the structure of the nerves, or soul, or both, which insure the possibility of reminiscence," &c. (p. 73), by what act is this possibility realized if not by the faculty of memory as ordinarily under-

* Carpenter, *Animal Physiology*, 383.

stood, or by the operation of that faculty, whatever it may be, by means of which they were originally perceived and preserved? That faculty may be called into activity by a new impression, by a simple suggestion, by a distinct effort of the will, but the recalling within the sphere of consciousness must be held to be distinct from the thing recalled, and the state into which it is recalled, and from the new impression or suggestion or volition by which it is recalled. The notion broached (p. 94), that these residua are "permanent changes which are made in connexion with every mental effort in the substance of the brain; certain cells added to the texture which ever after remain as the material representatives of our ideas," is not objected to as materialistic or as fanciful, but because no proof nor probability exists of such addition, and if there did, we know that no cells remain permanently for that or for any other purpose. The foundation of a rational psychology upon physiology is indispensable, since instinct, appetite, or reason, are admitted to be connected with the nervous system, and to be influenced by all vital actions; but it is a mistaken view, if not a prostitution of such an attempt, to use the one chiefly to fill up gaps which are impassable and unbridgeable by the other, or to associate with the multiplication of cells the preservation of ideas: a process, the nature of which is doubtful, and which, even in the showing of the author, should be the last selected as an adjunct to fugitive textures, and which as a mere impetration must dwell within the region of consciousness. Dr. Morell, recoiling from innate faculties, holds that each mind-germ contains within it an individual nature and constitution, (pp. 112-113), which, when brought into contact with the world, must of necessity develop certain faculties. Here the individual nature corresponds to the faculty depending upon structure for which we contend, while the faculties which are of necessity developed correspond to the additions to experience, received through the instrumentality of these faculties when acted upon by other appropriate stimulus.

Although, in tracing the relationship between perception and ideas, Dr. Morell announces that there is no trace of such a thing as an innate idea (p. 160), he, to our surprise, adds (p. 161): "They are neither born with us, nor are they impressed upon us from without; they are simply the product of the mind's free activity, operating in connexion with the world around." And at p. 278, "If we go back to the most primitive facts of mind, we find that there are certain impulses and tendencies to action which are impressed upon the nervous system prior to all consciousness;" and "every individual has a peculiar form and degree of sensibility of his own, which is clearly born with him, and with the production or modification of which *personal* experience has had nothing whatever to do." These passages, we submit, appear to demonstrate that the laws of similarity, &c., are built upon a mental constitution; it may be defined an activity, an individuality or primitive type, which is part of our nature, and which if not innate in the sense of idea is innate in the sense of faculty. But at p. 281, the law of similarity is stated to "begin its operations *spontaneously;*" that is, distinct from any natural or inevitable affinity in the "like experiences which unconsciously melt together, and any repulsion by which unlike are held apart." But we are inclined to demand, is the act of comparison by which the likeness or unlikeness is detected the result merely of impressions coming fortuitously together, or does it reside in a faculty, an individuality, which exists previous to the meeting together, and in which resides the act of approximation?

After a wide stride it appears that there is a distinction between an *à priori* fact or condition underlying the mental process by which knowledge is obtained, and *à priori* knowledge itself. "Consequently, there may exist," as Locke has affirmed, "not only ideas from *sensation*, but ideas from *reflection, i.e.*, ideas arising from the inward observation which the mind can make of its own nature and operations." (p. 307.) It would be superfluous to carry this analysis further, for in various places, as well as in the analysis of will previously quoted, there is a distinct admission of "primordial powers" or faculties in relation to external objects which do not call these powers into existence, but which minister to their activity;—but in carrying out this view the speculation that the physical feeling of want—hunger, for instance—is synonymous with an instinct which prompts us to seek food, appears to be altogether untenable. There is a confusion of the sensation of hunger and the debility which attends abstinence and exhaustion, and the psychical appetite for food. The sensation is a modification of pain; the appetite is a desire for gratification; but although the one may be implanted in order to relieve the other, the link which connects them, whether it be of causation or not, altogether escapes our observation. The sensation must exist anterior to the propensity; while the propensity exercises its influence in the absence of the sensation, and the appetite may become extinct while the sensation is urgent.

In more advanced inquiries into the freedom of the will, the proposition occurs that "motive means a mental state which we can control by our spontaneity or personality" (p. 383); and hence, if we can control the motive we can control the volition. It might be inquired, what is *spontaneity?* The fact here enunciated is admitted as a characteristic perhaps of all, but assuredly of energetic minds; but by what mental process do we influence the motive, and, indirectly, the will? It must be in virtue of some force behind, and more potent than, and different from, that which is controlled. It cannot be by calling up residua, for the intention is not subdued, nor supplanted, nor displaced; it cannot be by any voluntary process, otherwise there would occur the anomaly of a will standing in antagonism to another will! Is the phenomenon explicable on the theory of separate faculties or tendencies, occupying consciousness alternately, and urging action in different directions? For example, (p. 365) Dr. Morell writes, "every one is more or less conscious of the *antagonism* which sometimes shows itself between the reason and the will. Cases perpetually occur in which passion impels towards one course of action, while reason as decidedly points to another," &c. This is an admission of opposition between two powers; and the conclusion is fatal to the theory of development by laws. "Intelligence, then, is a *condition* essential to the existence of will, though it is not a *measure* of it."

We have differed from our author widely and earnestly, and have given a more careful examination of his work, chiefly because we regard him as demanding and deserving attention, whatever may be his teaching. The merit of such a work is less as a monograph than to make people think, and to show them the modes in which they think. Self-analysis is perhaps the characteristic and the blemish of thoughtful men in our time; but if such inquirers crave a formula, they cannot desire one clearer or more rudimentary of the kind, than that furnished in the "Introduction to Mental Philosophy on the Inductive Method."

---

